# Cardiometabolic risk factors among children and adolescents with overweight and Class 1 obesity: A cross-sectional study. Insights from stratification of Class 1 obesity

**DOI:** 10.3389/fendo.2023.1108618

**Published:** 2023-01-30

**Authors:** Afif Nakhleh, Rizan Sakhnini, Eyal Furman, Naim Shehadeh

**Affiliations:** ^1^ Maccabi Healthcare Services, Haifa, Israel; ^2^ The Azrieli Faculty of Medicine, Bar-Ilan University, Safed, Israel; ^3^ Ruth & Bruce Rappaport Faculty of Medicine, Technion, Israel Institute of Technology, Haifa, Israel

**Keywords:** cardiometabolic risk factors, children, adolescents, overweight, Class 1 obesity

## Abstract

**Context:**

Severe childhood obesity is associated with increased prevalence of cardiometabolic risk factors (CMRFs). Among children with Class 1 obesity, higher BMI may indicate greater cardiometabolic risk. Class 1 obesity reflects a wide spectrum of BMI values. Each 10% increase in BMI above the 95th percentile is equivalent to an average increase of 2.15 kg/m2 and 2.75 kg/m2 in BMI among children and adolescents, respectively. Such increments may be of clinical importance.

**Objectives:**

The study aimed to determine the prevalence and clustering of CMRFs in children and adolescents with BMI 110%-119% of the 95th BMI percentile.

**Methods:**

A cross-sectional analysis of data, from an Israeli health maintenance organization, of children and adolescents (5-17 years) with overweight or Class 1 obesity, and at least one measurement of lipid profile during Jan/2020-May/2021. CMRFs were defined as abnormal lipid profile, elevated alanine aminotransferase, hypertension, and prediabetes or diabetes. Study groups included overweight and Class 1 Obesity-A (BMI < 110%) and Obesity-B (BMI ≥ 110%) of the 95th BMI percentile.

**Results:**

Of 7211 subjects included, 40.2% were overweight, 50.3% obesity-A, and 9.5% obesity-B. Multivariable analyses showed that children and adolescents from the Obesity-B group had increased odds for higher triglycerides, LDL cholesterol, and ALT levels; and lower HDL cholesterol levels, as compared to Obesity-A. The odds of prediabetes (insignificant) tended to be higher in the Obesity-B group, which was associated with increased CMRFs clustering.

**Conclusions:**

Among children and adolescents with Class 1 obesity, BMI ≥ 110% of the 95th percentile was associated with higher prevalence and clustering of CMRFs.

## 1 Introduction

Obesity among children and adolescents has become a major public health challenge in the twenty-first century. Over the past decade, the prevalence of pediatric obesity in the United States increased from 17.7% to 21.5% (1). Pediatric obesity leads to a significant increase in cardiometabolic morbidity, including type 2 diabetes, hypertension, dyslipidemia, fatty liver, and cardiovascular complications (2–4). Screening tests for children with obesity include fasting lipids, glycated hemoglobin (HbA1c), fasting or random plasma glucose, and liver enzymes (5).

The clustering of cardiometabolic risk factors (CMRFs) in early childhood is of particular concern, given that the majority of children remain obese in adulthood (3). Skinner et al., using National Health and Nutrition Examination Survey (NHANES) data, demonstrated that severe obesity in children and adolescents was associated with increased prevalence of CMRFs, particularly among males (6). They also showed that, as expected, subjects with Class 1 obesity, defined as having a BMI ≥95th percentile to <120% of the 95th percentile for age and sex, had a higher risk of having most risk factors as compared to those with overweight (6, 7).

Each 10% increase in BMI above the 95th percentile is equivalent to an average increase of 2.1 kg/m^2^ and 2.7 kg/m^2^ in BMI among male children and adolescents, respectively; and of 2.2 kg/m^2^ and 2.8 kg/m^2^ among female children and adolescents, respectively (**Table S1**) (8). Such increments in BMI may be clinically significant. For example, the Princeton Follow-up Study demonstrated that the risk of metabolic syndrome in adulthood increased by 24% for each 10% increase in age-specific BMI in children and youth (9).

The question of whether the increment in BMI above 109% of the 95th percentile translates into a significant difference in the risk of cardiometabolic morbidity within the Class 1 obesity group has not yet been addressed. Therefore, in the present study, we aimed at assessing the prevalence and clustering of CMRFs in Israeli children and adolescents with a BMI of 110%-119% of the 95th BMI percentile, as compared to those with overweight and Class 1obesity.

### 2 Materials and methods

This non-interventional, cross-sectional analysis of data was conducted using the electronic medical database of Maccabi Healthcare Services (MHS), a large health maintenance organization in Israel serving over two million patients. All data were collected from the MHS automated database during May 2021. Clinical data included (1): demographic and anthropometric information on age, gender, socioeconomic status (SES) of the place of residence, weight, BMI percentile, and blood pressure (2); laboratory results including fasting serum total cholesterol, LDL cholesterol, HDL cholesterol, triglycerides, plasma glucose, glycated hemoglobin (HbA1c), and alanine aminotransferase (ALT); and (3) data from the MHS diabetes registry. The retrieved data included the last available value in the database recorded during January 2020 - May 2021, except for blood pressure for which we included the last two measurements when available, and fasting plasma glucose and HbA1c for which we retrieved all the available data.

We obtained approval from the MHS institutional review board and ethics committee to access and analyze data. Individual patient informed consent was not required because of the anonymized nature of patient records.

### 2.1 Study sample and definitions

We performed a cross-sectional analysis of data from children and adolescents 5-17 years of age with overweight or Class 1 obesity, who had at least one measurement of lipid profile during the study period.

Age was expressed in years and rounded to the nearest year (cutoff points are at the midpoint of the child’s year). BMI was classified according to percentiles established by the Centers for Disease Control and Prevention (CDC). These were validated for Israeli children and adolescents, for age (by month) and sex (10). Overweight was defined as a BMI between the 85th and 94th percentile. Obesity-A was defined as a BMI at or greater than the 95th percentile up to 109% of the 95th percentile. A BMI between 110% and 119% of the 95th percentile was defined as Obesity-B. Subjects with a BMI at or greater than 120% of the 95th percentile (or BMI greater than 35 kg/m^2^) were not included in this study.

Data were obtained on the socioeconomic status (SES) of the place of residence based on the Israeli Central Bureau of Statistics scoring system: low (score 1-4 out of 10), medium (score 5-8 out of 10), and high (score 9-10 out of 10).

Of 26783 children and adolescents (14744 females and 12039 males) aged 5-17 years with documented BMI ≥85th percentile between January 2020 through May 2021, 7211 subjects had a BMI<120% of the 95th percentile and available lipid profile data and thus were included in the study (**Figure S1**).

We aimed at evaluating children or adolescents for potential comorbidities associated with overweight and obesity. **Table 1** summarizes characteristics of the sample age groups and the definition of comorbidities and abnormal values for cardiometabolic variables.

**Table 1:** Definitions of comorbidities and abnormal values for cardiometabolic variables.

Variable	Age group, yr	Number of subjects evaluated	Definition of comorbidity or abnormal value
**Total cholesterol**	5-17	7211	≥200 mg/dL
**LDL cholesterol**	5-17	7211	≥130 mg/dL
**HDL cholesterol**	5-17	7211	≤35 mg/dL
**Triglycerides**	5-17	7211	≥150 mg/dL
**ALT**	5-17	6869	>25 U/L (boys) >22 U/L (girls)
**Prediabetes**	5-17	7211	≥2 FPG tests 100-125 mg/dL or ≥1 HbA1c test 5.7%-6.4%
**Diabetes mellitus**	5-17	7211	Inclusion in MHS diabetes registry
**Systolic BP**	5-12	1226	≥95^th^ percentile (adjusted to the 50th percentile of age- and gender-specific height)
	13-17	3773	≥130 mm Hg
**Diastolic BP**	5-12	1226	≥95^th^ percentile (adjusted to the 50th percentile of age- and gender-specific height)
	13-17	3773	≥80 mm Hg

ALT, alanine aminotransferase; BP, blood pressure; FPG, fasting plasma glucose; HbA1c, glycated hemoglobin; HDL, high-density lipoprotein; LDL, low-density lipoprotein; MHS, Maccabi Healthcare Services.

Abnormal lipid profile values were defined using standard cutoff values for levels of total cholesterol (≥200 mg/dL), LDL cholesterol (≥130 mg/dL), HDL cholesterol (<35 mg/dL), and triglycerides (≥150 mg/dL). Serum ALT concentrations above the 95th percentile (>25 U/L for boys and >22U/L for girls) were regarded as abnormal, as proposed by Schwimmer JB et al. (11). In our sample, 6869 children and adolescents had at least one documented ALT value.

Blood pressure was recorded as the mean value of up to two measurements or as a single measurement (3810 of the children and adolescents had two measurements, 1189 had only one measurement, and 2212 had no measurement). For children aged 5–12 years, we used standardized blood pressure tables in which abnormal values were determined according to age and gender and adjusted to the 50th percentile of height; abnormal values were defined as any value at or above the 95th percentile in **Table S2**. For children aged ≥13 years, we used cutoffs of ≥130 mm Hg for systolic blood pressure and ≥80 mm Hg for diastolic blood pressure (12).

We defined individuals as having prediabetes if they fulfilled the following criteria: at least two separate fasting plasma glucose values between 100 mg/dL and 125mg/dL, or a single measurement of HbA1c between 5.7% and 6.4%; and the person was not included in the MHS diabetes registry. We used data from MHS automated patients’ registry to diagnose diabetes (13). This registry is based on a validated algorithm that collects data from electronic medical records, laboratory results (HbA1c and fasting plasma glucose), dispensed medications (oral glucose-lowering agents or insulin), and clinical diagnoses (more details are available in the **Supplementary file**: **Methods**).

### 2.2 Statistical analysis

Continuous data are presented as mean ± standard deviation (SD) or median (interquartile range). Categorical data are presented as absolute numbers and percentages. Prevalence is reported as a percentage with a 95% confidence interval.

The chi-square test or Fisher’s exact test was used to assess the association between categorical variables. T-test or ANOVA were used to assess the association between continuous variables.

To examine the effect of weight on CMRFs we performed multivariable analyses using generalized linear models (GLMs) with a logarithmic link. We reported the odds ratios (after exponentiation of the coefficients). We also performed logistic regression analyses to evaluate the effect of weight on the clustering of CMRFs. Models were adjusted when appropriate for age and sex. A p-value < 0.05 was considered significant.

All statistical analyses were performed using STATA, version 17 (Stata Corp., Texas, USA).

## 3 Results

Among 7211 children and adolescents with a BMI at the 85th percentile or higher, 40.2% were classified as overweight, 50.3% as obesity-A, and 9.5% as obesity-B (**Table 2**). **Figure S2** depicts median BMI values classified by weight categories and age. No significant correlation was found between SES and severity of obesity (**Table S3**).

**Table 2:** Distribution of weight groups by age and sex.

	Overweight	Obesity-A	Obesity-B	Total
All subjects (5-17) n, %	2902 (40.2)	3624 (50.3)	685 (9.5)	7211 (100)
Male	1086 (37.4)	1506 (41.6)	294 (42.9)	2886 (40)
Female	1816 (62.6)	2118 (58.4)	391 (57.1)	4325 (60)
Children (5-11) n, %	489 (33.3)	806 (54.9)	174 (11.8)	1469 (20.4)
Male	175 (35.8)	280 (34.7)	63 (36.2)	518 (35.3)
Female	314 (64.2)	526 (65.3)	111 (63.8)	951 (64.7)
Adolescents (12-17) n, %	2413 (42)	2818 (49.1)	511 (8.9)	5742 (79.6)
Male	911 (37.8)	1226 (43.5)	231 (45.2)	2368 (41.2)
Female	1502 (62.2)	1592 (56.5)	280 (54.8)	3374 (58.8)

**Table S4** shows the mean values for each cardiometabolic variable for all subjects. These values increased with the severity of obesity, except for HDL cholesterol.

**Figure 1:**
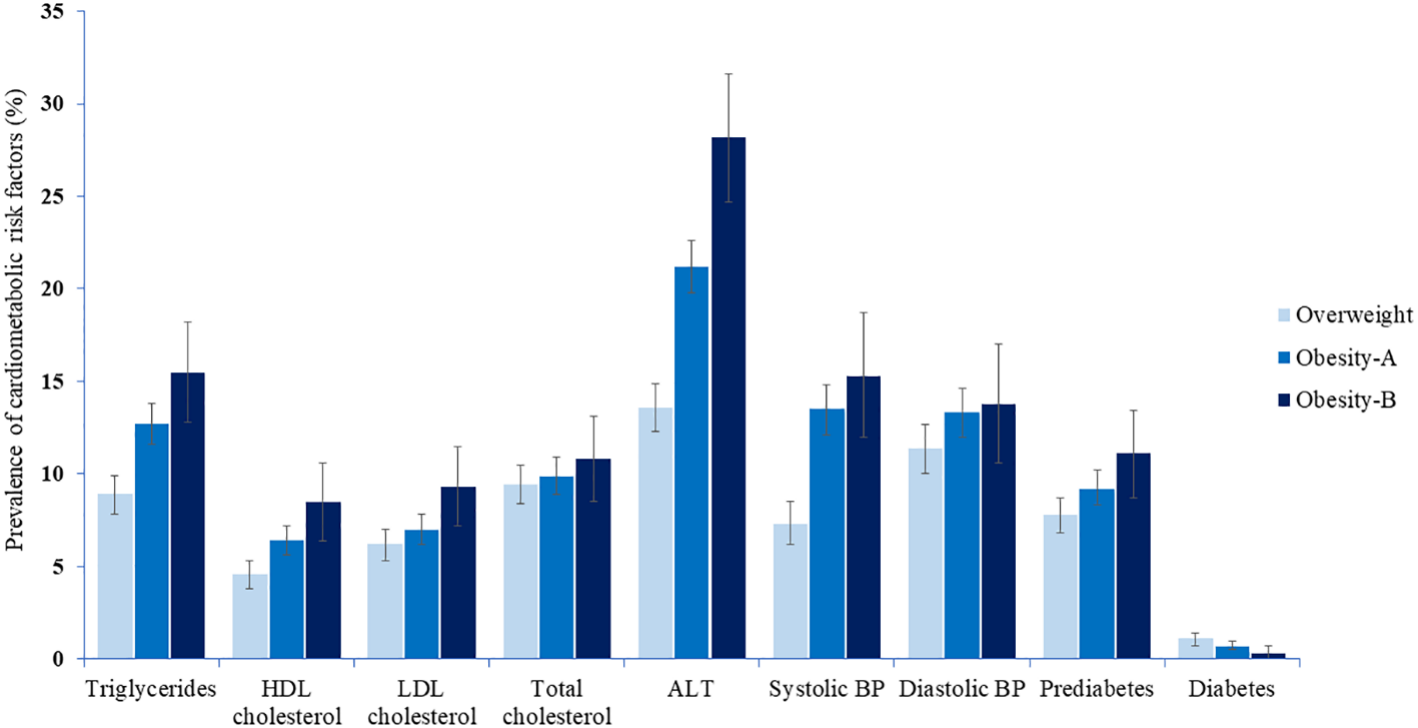
The prevalence of cardiometabolic risk factors among children and adolescents by weight category.

**Table 3** and **Figure 1** show the prevalence of CMRFs among children and adolescents, classified by weight category. Except for total cholesterol, diastolic blood pressure, and diabetes, the prevalence of CMRFs (including prediabetes) consistently increased with the severity of obesity in adolescents (**Tables 3**, **S5**). This trend was evident albeit to a lesser extent among children for whom only systolic blood pressure and ALT levels differed significantly between weight groups (**Tables 3**, **S6**).

**Table 3:** Prevalence of comorbidities and abnormal values for cardiometabolic variables by weight among children and adolescents.

Risk factor variable and weight category	All subjects			Children			Adolescents		
	Subjects, n	Prevalence, % (95% CI)	P value	Subjects, n	Prevalence, % (95% CI)	P value	Subjects, n	Prevalence, % (95% CI)	P value
Triglycerides			<0.001			0.11			<0.001
Overweight	2902	8.9(7.8-9.9)		489	7.4 (5-9.7)		2413	9.2 (8-10.3)	
Obesity-A	3624	12.7 (11.6-13.8)		806	9.2 (7.2-11.2)		2818	13.7 (12.4-15)	
Obesity-B	685	15.5 (12.8-18.2)		174	12.6 (7.7-17.6)		511	16.4 (13.2-19.7)	
HDL cholesterol			<0.001			0.62			<0.001
Overweight	2902	4.6 (3.8-5.3)		489	1.8 (0.6-3)		2413	5.1 (4.3-6)	
Obesity-A	3624	6.4 (5.6-7.2)		806	2.6 (1.5-3.7)		2818	7.5 (6.5-8.5)	
Obesity-B	685	8.5 (6.4-10.6)		174	2.9 (0.4-5.4)		511	10.4 (7.7-13)	
LDL cholesterol			0.01			0.55			0.015
Overweight	2902	6.2 (5.3-7)		489	7.2 (4.9-9.4)		2413	6 (5-6.9)	
Obesity-A	3624	7 (6.2-7.8)		806	6.8 (5.1-8.6)		2818	7.1 (6.1-8)	
Obesity-B	685	9.3 (7.2-11.5)		174	9.2 (4.9-13.5)		511	9.4 (6.9-11.9)	
Total cholesterol			0.54			0.33			0.57
Overweight	2902	9.4 (8.4-10.5)		489	12.7 (9.7-15.6)		2413	8.8 (7.7-9.9)	
Obesity-A	3624	9.9 (8.9-10.9)		806	10.8 (8.7-12.9)		2818	9.6 (8.5-10.7)	
Obesity-B	685	10.8 (8.5-13.1)		174	14.4 (9.2-19.6)		511	9.6 (7-12.1)	
Systolic BP			<0.001			<0.001			<0.001
Overweight	2059	7.3 (6.2-8.5)		215	11.2 (7-15.4)		1844	6.9 (5.7-8)	
Obesity-A	2490	13.5 (12.1-14.8)		339	23.3 (18.8-27.8)		2151	11.9 (10.5-13.3)	
Obesity-B	450	15.3 (12-18.7)		70	25.7 (15.5-36)		380	13.4 (10-16.8)	
Diastolic BP			0.10			0.28			0.28
Overweight	2059	11.4 (10-12.7)		215	11.2 (7-15.4)		1844	11.4 (9.9-12.8)	
Obesity-A	2490	13.3 (12-14.6)		339	15.9 (12-19.8)		2151	12.9 (11.5-14.3)	
Obesity-B	450	13.8 (10.6-17)		70	15.7 (7.2-24.2)		380	13.4 (10-16.8)	
Prediabetes			<0.001			0.61			<0.001
Overweight	2902	7.8 (6.8-8.7)		489	3.5 (1.9-5.1)		2413	8.6 (7.5-9.7)	
Obesity-A	3624	9.2 (8.3-10.2)		806	3.1 (1.9-4.3)		2818	11 (9.8-12.1)	
Obesity-B	685	11.2 (8.7-13.4)		174	4.6 (1.5-7.7)		511	13.3 (10.4-16.3)	
Diabetes			0.09			0.87			0.22
Overweight	2902	1.1 (0.7-1.4)		489	0.6 (0-1.3)		2413	1.2 (0.7-1.6)	
Obesity-A	3624	0.7 (0.5-1)		806	0.5 (0-1)		2818	0.8 (0.5-1.1)	
Obesity-B	685	0.3 (0-0.7)		174	0		511	0.4 (0-0.9)	
ALT			<0.001			<0.001			<0.001
Overweight	2783	13.6 (12.3-14.9)		471	15.3 (12-18.5)		2312	13.3 (11.9-14.7)	
Obesity-A	3440	21.2 (19.8-22.6)		759	22.7 (19.7-25.6)		2681	20.8 (19.2-22.3)	
Obesity-B	646	28.2 (24.7-31.6)		165	30.9 (23.9-38)		481	27.2 (23.3-31.2)	

P value for comparison among three groups. A p value < 0.05 was considered significant.

ALT, alanine aminotransferase; BP, blood pressure; FPG, fasting plasma glucose; HbA1c, glycated hemoglobin; HDL, high-density lipoprotein; LDL, low-density lipoprotein.

**Table 4** shows a direct comparison of CMRFs between weight groups of children and adolescents in multivariable generalized linear models that adjusted for age and sex. Children and adolescents in the obesity-B group had greater odds for higher triglycerides, LDL cholesterol, and ALT and lower HDL cholesterol levels, as compared to the obesity-A group. These differences were significant in females except for the difference in ALT levels, the only observation that was significant in males. The odds of prediabetes tended to be higher in the obesity-B group but did not reach significance. Overweight children and adolescents had lower odds for most of the CMRFs.

**Table 4:** Odds ratios for cardiometabolic risk factors among children and adolescents by sex and weight.

Risk factor variable and weight category	All subjects			Female subjects			Male subjects		
	Subjects, n	Odds ratio, (95% CI)	P value	Subjects, n	Odds ratio, (95% CI)	P value	Subjects, n	Odds ratio, (95% CI)	P value
Triglycerides									
Overweight	2902	0.66 (0.56-0.78)	<0.001	1816	0.75 (0.61-0.92)	0.006	1086	0.51 (0.39-0.68)	<0.001
Obesity-A	3624	Reference		2118	Reference		1506	Reference	
Obesity-B	685	1.28 (1.01-1.61)	0.04	391	1.36 (1.01-1.82)	0.04	294	1.16 (0.80-1.69)	0.42
HDL cholesterol									
Overweight	2902	0.66 (0.53-0.83)	<0.001	1816	0.69 (0.50-0.96)	0.03	1086	0.65 (0.48-0.89)	0.006
Obesity-A	3624	Reference		2118	Reference		1506	Reference	
Obesity-B	685	1.44 (1.06-1.95)	0.02	391	1.65 (1.07-2.53)	0.02	294	1.24 (0.80-1.91)	0.32
LDL cholesterol									
Overweight	2902	0.86 (0.71-1.05)	0.15	1816	0.96 (0.75-1.23)	0.79	1086	0.69 (0.49-0.97)	0.03
Obesity-A	3624	Reference		2118	Reference		1506	Reference	
Obesity-B	685	1.37 (1.03-1.83)	0.03	391	1.49 (1.03-2.15)	0.03	294	1.20 (0.76-1.92)	0.42
Total Cholesterol									
Overweight	2902	0.94 (0.80-1.11)	0.49	1816	1.01 (0.83-1.24)	0.88	1086	0.77 (0.57-1.05)	0.09
Obesity-A	3624	Reference		2118	Reference		1506	Reference	
Obesity-B	685	1.10 (0.85-1.43)	0.47	391	1.22 (0.87-1.70)	0.21	294	0.92 (0.58-1.46)	0.725
Systolic BP									
Overweight	2059	0.56 (0.46-0.67)	<0.001	1282	0.58 (0.45-0.75)	<0.001	777	0.53 (0.41-0.69)	<0.001
Obesity-A	2490	Reference		1413	Reference		1077	Reference	
Obesity-B	450	1.11 (0.85-1.44)	0.43	251	1.29 (0.91-1.85)	0.15	199	0.94 (0.64-1.38)	0.75
Diastolic BP									
Overweight	2059	0.78 (0.66-0.92)	0.003	1282	0.84 (0.69-1.04)	0.10	777	0.71 (0.55-0.92)	0.01
Obesity-A	2490	Reference		1413	Reference		1077	Reference	
Obesity-B	450	0.99 (0.76-1.28)	0.96	251	1.10 (0.78-1.54)	0.59	199	0.85 (0.56-1.28)	0.43
Prediabetes									
Overweight	2902	0.76 (0.64-0.91)	0.003	1816	0.78 (0.61-0.98)	0.04	1086	0.76 (0.58-1.01)	0.06
Obesity-A	3624	Reference		2118	Reference		1506	Reference	
Obesity-B	685	1.29 (0.99-1.69)	0.06	391	1.37 (0.96-1.96)	0.07	294	1.18 (0.78-1.78)	0.43
Diabetes									
Overweight	2902	1.59 (0.95-2.67)	0.08	1816	1.54 (0.80-2.95)	0.20	1086	1.67 (0.72-3.89)	0.23
Obesity-A	3624	Reference		2118	Reference		1506	Reference	
Obesity-B	685	0.40 (0.10-1.71)	0.22	391	0.34 (0.04-2.55)	0.29	294	0.51 (0.07-4.00)	0.52
ALT									
Overweight	2783	0.59 (0.52-0.68)	<0.001	1735	0.56 (0.47-0.68)	<0.001	1048	0.63 (0.52-0.77)	<0.001
Obesity-A	3440	Reference		2007	Reference		1433	Reference	
Obesity-B	646	1.42 (1.18-1.72)	<0.001	368	1.28 (0.98-1.67)	0.07	278	1.61 (1.22-2.12)	<0.001

GLMs that controlled for age and sex were used for these analyses. Obesity-A is the referent group.

A p value < 0.05 was considered significant.

ALT, alanine aminotransferase; BP, blood pressure; FPG, fasting plasma glucose; HbA1c, glycated hemoglobin; HDL, high-density lipoprotein; LDL, low-density lipoprotein.

**Figure 2:**
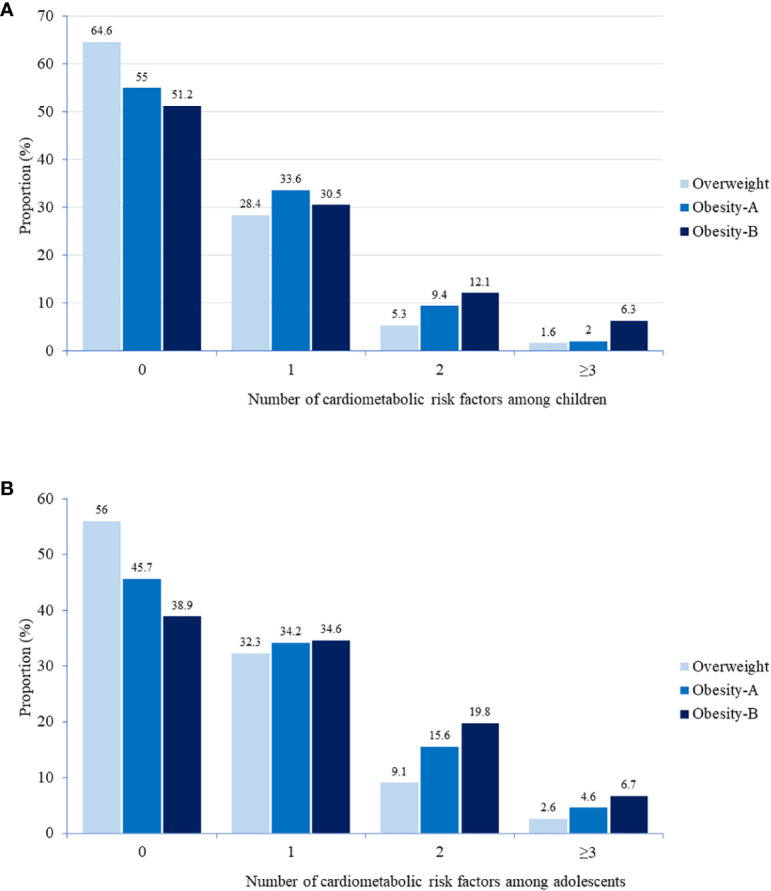
Distribution of cardiometabolic risk factors among children **(A)** and adolescents **(B)** by weight category. Cardiometabolic risk factors include hyperglycemia (prediabetes or diabetes), high triglycerides, LDL cholesterol, ALT, and systolic or diastolic BP, or low HDL cholesterol. The number of cardiometabolic risk factors increased with the severity of obesity in children (p<0.001) and adolescents (p<0.001). P value for difference between groups was determined by chi square test.

The number of CMRFs increased with the severity of obesity in children and adolescents (**Table S7**; **Figure 2**). The prevalence of clustering of ≥2 and ≥3 CMRFs were 12.1% and 6.3%, respectively; and 19.8% and 6.7% among children and adolescents from the obesity-B group, respectively (**Figure 2**). **Table 5** shows the adjusted odds ratios for the number of CMRFs across weight groups of children and adolescents. Compared with the obesity-A group, subjects from the obesity-B group were at 52% and 76% higher risk of clustering of ≥ 2 and ≥3 CMRFs, respectively (**Table 5**).

**Table 5:** Odds ratios of having ≥1, ≥2, and ≥3 (vs zero) cardiometabolic risk factors* by weight category in children and adolescents.

Weight category	≥1 cardiometabolic risk factors		≥2 cardiometabolic risk factors		≥3 cardiometabolic risk factors	
	Odds ratio, (95% CI)	P value	Odds ratio, (95% CI)	P value	Odds ratio, (95% CI)	P value
**Overweight**	0.64 (0.58-0.71)	<0.001	0.51 (0.44-0.59)	<0.001	0.56 (0.42-0.75)	<0.001
**Obesity-A**	Reference		Reference		Reference	
**Obesity-B**	1.29 (1.09-1.53)	0.002	1.52 (1.24-1.85)	<0.001	1.76 (1.25-2.49)	0.001

Cardiometabolic risk factors include hyperglycemia (prediabetes or diabetes), high triglycerides, LDL cholesterol, ALT, and systolic or diastolic BP or low HDL cholesterol.

Logistic regression that controlled for age and sex was used for these analyses. Obesity-A is the referent group.

A p value < 0.05 was considered significant.

ALT, alanine aminotransferase; BP, blood pressure; HDL, high-density lipoprotein; LDL, low-density lipoprotein.

## 4 Discussion

The present study shows a higher prevalence of most of the cardiovascular risk factors in children and adolescents with a BMI between 110%-119% of the 95th BMI percentile (obesity-B group) as compared to individuals with lower degree of obesity. This ten percent increment in BMI within the Class 1 obesity group translates into significantly higher odds for clustering of CMRFs.

Severe obesity in childhood is associated with significant metabolic and cardiovascular morbidity (14). In our study, we aimed at assessing children and adolescents with lower degrees of obesity. Class 1 obesity reflects a wide spectrum of BMI values. Each 10% increase in BMI above the 95th percentile is equivalent to an average increase of 2.15 kg/m^2^ and 2.75 kg/m^2^ in BMI among children and adolescents, respectively (**Table S1**) (8). Such increments may be of high clinical importance. For example, the Princeton Follow-up Study demonstrated that the risk of metabolic syndrome in adulthood increased by 24% for each 10% increase in age-specific BMI in childhood (9). In young adults, each 1-kg/m^2^ increase in BMI was associated with a 6% higher risk of developing type 2 diabetes before the age of 45 years (15). In a cohort of Danish and Finnish subjects, each z-score increase in BMI at 7 years of age (equivalent to a 1.5 to 2.5 kg/m^2^ increment) was associated with a 5%-10% greater risk of coronary heart disease in adulthood (16). Moreover, the risk of mortality increases significantly throughout the overweight and obesity range. In adults younger than 50 years of age, every five units higher BMI above 25 kg/m^2^ was associated with an approximate 52% higher risk of premature death (17). These findings emphasize the importance of losing relative weight at a young age.

We subclassified Class 1 obesity into two groups to “zoom in” and better understand the cardiometabolic morbidity associated with lower degrees of obesity in childhood.

In our study, the prevalence of most CMRFs increased with the severity of obesity, except for diastolic blood pressure, diabetes, and total cholesterol (**Table 3**).

Our results are in line with Sorof et al. that showed a progressive increase in systolic blood pressure with each increase in BMI percentile, whereas diastolic blood pressure showed no association (18). In our study, the prevalence of prediabetes was higher in the obesity groups as expected. However, the prevalence of diabetes was low with no difference seen between the groups. These registry-derived diagnoses include patients with all forms of diabetes, including type 1 diabetes, which might explain the higher (though insignificant) trend of having diabetes in the overweight group (**Table 4**). However, Twig et al. showed that the cumulative incidence of early-onset type 2 diabetes was more than 2-fold higher among adolescents with severe obesity than among those with Class 1 obesity (4). This relationship should be regarded as a continuum in which each increment above the 95th BMI increases the risk of prediabetes and the transition toward type 2 diabetes.

Obesity is associated with elevated ALT levels. We defined abnormal serum ALT concentrations as >25 U/L for boys and >22U/L for girls (11). This is because liver biopsy specimens from children with normal or mildly elevated ALT (≥26 to 50 U/L for boys and ≥23 to 44 U/L for girls) were histologically abnormal, including advanced fibrosis (19). In our study, an increment in BMI above 109% of the 95th percentile more than doubled the risk for abnormal ALT values in children and adolescents as compared to those with overweight (**Table S5**, **S6**). Despite being nonspecific when used alone, the increased plasma concentrations of ALT with higher severity of obesity might reflect the presence of nonalcoholic fatty liver disease (NAFLD) in a substantial proportion of these children.

There were differences observed between children and adolescents in terms of their lipid and glucose profiles, with children showing nonsignificant differences across the weight groups (**Table 3**). This suggests that the duration of obesity may play a stronger role than BMI alone.

There were important differences between male and female subjects in our study. It should be noted that girls constitute 60% of the study sample, although the prevalence of obesity in the general population is greater among boys (20, 21). Given the cross-sectional nature of the study and the eligibility for inclusion based on available data, this gender disparity may have occurred by chance. The present study demonstrates clearly that an increment in BMI above 109% of the 95th percentile is clinically significant, particularly among females. This increment increases the odds of higher triglycerides and LDL cholesterol and lower HDL cholesterol levels as compared to in females with lower degree of obesity (**Table 4**). This markedly unfavorable lipid profile relative to weight gain in girls has been reported in a previous study which demonstrated slightly stronger indirect effects of weight gain, through childhood adiposity, in girls as compared with boys (22). There was a nonsignificant trend towards increased odds for prediabetes and higher ALT levels in females with a BMI above 109% of the 95th percentile as compared to those with lower degree of obesity. In males, an increment above 109% of the 95th BMI percentile was associated with increased odds for higher ALT levels. This result is in line with previous studies showing that prevalence was generally higher in boys as compared with girls and increased incrementally with greater BMI (23, 24).

Our findings differ from those in other reports. Some studies showed minimal differences between boys and girls and another study (in which 16.7% of the sample had severe obesity) showed a higher prevalence of risk factors in males (6, 25–27).

The clustering of CMRFs in childhood is of high concern, taking into account the fact that around 80% of them remain obese in adulthood (3). Indeed, this observation justifies the evaluation of cardiometabolic variables at a young age, especially among those who are overweight or obese.

Several definitions of metabolic syndrome in children and adolescents have been proposed (28–33). These definitions are diverse and lack uniformity, and there is no clear consensus on which to use. The American Academy of Pediatrics recommends shifting the focus to the concept of clustering of CMRFs rather than defining metabolic syndrome in children and adolescents (34).

In the Bogalusa Heart Study, the clustering of CMRFs was associated with increased severity of asymptomatic coronary atherosclerotic lesions in young people (35). Since the clustering of risk factors is present in childhood and continues into young adulthood, the presence of multiple risk factors such as hyperglycemia, hypertension, and abnormal lipid profile may indicate a faster progression of atherosclerosis in young people.

Using conservative thresholds for prediabetes, dyslipidemia, and hypertension, a large multi-ancestral cohort demonstrated a direct correlation between the severity of obesity and prevalence of CMRFs, whereby each half-unit increase in the BMI z score increased the risk of having cardiovascular risk factors’ clustering by 55% (29). Skinner et al. showed that values for some, but not all, CMRFs were higher with increased severity of obesity in children and adolescents, and demonstrated that greater severity of obesity is associated with a higher risk of lower HDL-cholesterol levels, elevated plasma triglycerides, and high systolic and diastolic blood pressure (6).

In our study, subjects with BMI between 110%-120% of the 95th BMI percentile had a higher prevalence of CMRFs clustering (17.8% and 6.6% for clustering of ≥2 and ≥3 risk factors, respectively) (**Table S7**). They exhibited a substantially higher risk for CMRFs clustering as compared to those with lower degree of obesity (**Table 5**). This highlights the need for further stratification of weight groups at a young age to better reflect the risk for metabolic and cardiovascular morbidity.

Previous research has shown that a lower SES during childhood is linked to a higher risk of developing metabolic syndrome in adulthood, even when controlling for other childhood risk factors (36). However, our study did not find a significant association between SES and the level of obesity, thus it cannot be considered a confounding factor.

There are several limitations to our study that should be considered. First, this is a cross-sectional study, which cannot prove the causality between obesity and CMRFs. In addition, we cannot exclude any secondary causes of obesity, such as genetic or hormonal factors. Second, we lacked data on lifestyle, physical activity, and other indicators of obesity such as waist circumference. The latter may be more sensitive than BMI in the context of this study and better define cardiovascular risk in the long term. Third, our sample might differ from the general population in terms of gender representation, as obesity is more common in boys (20). A considerable difference was observed in the number of individuals with obesity-A and obesity-B (50.3% compared to 9.5%). It should be noted that our sample was limited to individuals with available lipid profile data. This limitation, combined with the observational nature of the study, raises the possibility that the representation of obesity-B in our sample may not accurately reflect its prevalence in the general population. Finally, the sample size was relatively small in weight subgroups of children younger than 12 years old, which resulted in some estimates with wide confidence intervals, deeming them insignificant. Thus, some of these estimates should be interpreted with caution.

Our study has certain strengths. The study was based on a relatively large sample of children and adolescents. This permitted the evaluation of a wide variety of CMRFs and the exploration of their clustering among weight subgroups. In conclusion, among children and adolescents with Class 1 obesity, a BMI ≥ 110% of the 95th percentile was associated with a higher prevalence of and greater clustering of CMRFs. The consideration of this group (obesity-B group) in the standard obesity classification may assist in the identification of children and adolescents who could be at a greater risk for abnormal lipid profile, hyperglycemia, and abnormal ALT levels.

## Data availability statement

The raw data supporting the conclusions of this article will be made available by the authors, without undue reservation.

## Ethics statement

The studies involving human participants were reviewed and approved by Maccabi Healthcare Services institutional review board and ethics committee. Approval number 0183-20-MHS. Written informed consent from the participants’ legal guardian/next of kin was not required to participate in this study in accordance with the national legislation and the institutional requirements.

## Author contributions

AN and RS were responsible for the design of the study and data acquisition. AN and RS contributed to the analysis and the interpretation of data, drafted the manuscript and revised it critically for important intellectual content. NS and EF contributed to the analysis and the interpretation of data and revised the manuscript critically for important intellectual content. All authors contributed to the article and approved the submitted version.
