# Supplementary material for: Cardiometabolic risk factors among children and adolescents with overweight and Class 1 obesity: A cross-sectional study. Insights from stratification of Class 1 obesity
Source: Front Endocrinol (Lausanne). 2023 Jan 30;14:1108618. doi: 10.3389/fendo.2023.1108618 (PMC9927000; doi:10.3389/fendo.2023.1108618)
Supplement: Supplementary file 1 [file Table_1.docx]

Supplementary Material

Cardiometabolic risk factors among children and adolescents with overweight and Class 1 obesity: a cross-sectional study. Insights from stratification of Class 1 obesity.

Afif Nakhleh, Rizan Sakhnini, Eyal Furman, Naim Shehadeh

**Correspondence:**

Afif Nakhleh

Diabetes and Endocrinology Clinic, Maccabi Healthcare Services. Simcha Golan Road 54, Haifa 3299001, Israel. The Azrieli Faculty of Medicine, Bar-Ilan University, Safed, Israel. E-mail: [anakhleh@gmail.com](mailto:anakhleh@gmail.com) Tel: +972-54-5958840. ORCID ID: 0000-0003-1206-1516

1. **Supplementary Data to Methods:**

Inclusion in the diabetes registry required at least one of the following five criteria:

1. **Diagnoses:** Active diagnosis or a visit diagnosis made by a primary care physician, a diabetes expert, an endocrinologist, or an ophthalmologist. This should be accompanied by at least one of the following
   1. Measurement of HbA1c≥6.5% within 6 months before or after receiving the diagnosis
   2. Two fasting plasma glucose tests of ≥126 mg/dL, one within 6 months before or after receiving the diagnosis, and the other without time constraints.
2. **Insulin:** Two insulin purchases on two different dates within 3 months. The registry does not capture females aged 18-55 years, without any of the other criteria, a glucose tolerance test or glucose challenge test, or insulin purchases within a period shorter than 4 months.
3. **Oral glucose-lowering agents:** Two purchases on two different dates within 3 months, as well as an HbA1c measurement ≥6.5% and/or a fasting plasma glucose ≥126 mg/dL.
4. **HbA1c measurement:** Measurement of ≥7.25%.

**Plasma glucose measurement:** at least two measurements of ≥200mg/dL, at least 30 days apart

1. **Supplementary Tables**

**Table S1.** Cutoff points for BMI percentiles according to age and sex

|  | **BMI cutoff point, kg/m^2^**  **Males** | | | | **BMI cutoff point, kg/m^2^**  **Females** | | | |
| --- | --- | --- | --- | --- | --- | --- | --- | --- |
| **Age (Y^*^)** | **85th percentile** | **95^th^ percentile** | **110% of the 95th percentile** | **120% of the 95th percentile** | **85th percentile** | **95th percentile** | **110% of the 95th percentile** | **120% of the 95th percentile** |
| **5** | 16.9 | 18.1 | 19.9 | 21.8 | 16.9 | 18.5 | 20.4 | 22.2 |
| **6** | 17.2 | 18.8 | 20.7 | 22.5 | 17.3 | 19.2 | 21.1 | 23.1 |
| **7** | 17.7 | 19.6 | 21.6 | 23.5 | 18 | 20.2 | 22.2 | 24.2 |
| **8** | 18.3 | 20.6 | 22.7 | 24.7 | 18.7 | 21.2 | 23.3 | 25.5 |
| **9** | 19 | 21.6 | 23.8 | 25.9 | 19.5 | 22.4 | 24.6 | 26.9 |
| **10** | 19.8 | 22.7 | 25 | 27.2 | 20.4 | 23.6 | 26 | 28.3 |
| **11** | 20.6 | 23.7 | 26.1 | 28.5 | 21.3 | 24.7 | 27.2 | 29.6 |
| **12** | 21.4 | 24.7 | 27.2 | 29.7 | 22.2 | 25.8 | 28.4 | 30.9 |
| **13** | 22.3 | 25.6 | 28.2 | 30.7 | 23 | 26.8 | 29.5 | 32.1 |
| **14** | 23.1 | 26.5 | 29.2 | 31.7 | 23.7 | 27.7 | 30.5 | 33.2 |
| **15** | 23.8 | 27.2 | 29.9 | 32.6 | 24.4 | 28.5 | 31.4 | 34.2 |
| **16** | 24.6 | 27.9 | 30.7 | 33.5 | 24.9 | 29.3 | 32.2 | 35.1 |
| **17** | 25.3 | 28.6 | 31.5 | 34.3 | 25.4 | 30 | 33 | 36 |

***** Cutoff points are at the midpoint of the child's year (from the CDC growth charts (8)).

BMI, body mass index

**Table S2.** Hypertension definition

| **Age (Y)** | **SBP Male** | **DBP Male** | **SBP Female** | **DBP Female** |
| --- | --- | --- | --- | --- |
| **5** | 109 | 69 | 110 | 71 |
| **6** | 111 | 71 | 111 | 72 |
| **7** | 112 | 73 | 112 | 73 |
| **8** | 114 | 74 | 113 | 74 |
| **9** | 115 | 76 | 114 | 75 |
| **10** | 116 | 77 | 116 | 76 |
| **11** | 118 | 78 | 118 | 77 |
| **12** | 121 | 78 | 122 | 78 |
| **13** | 130 | 80 | 130 | 80 |
| **14** | 130 | 80 | 130 | 80 |
| **15** | 130 | 80 | 130 | 80 |
| **16** | 130 | 80 | 130 | 80 |
| **17** | 130 | 80 | 130 | 80 |

**SBP,** systolic blood pressure**; DBP,** diastolic blood pressure

**Table S3.** Distribution of weight groups by socioeconomic status of place of residence

| **Weight groups** | **SES groups n, %** | | | **P-value** |
| --- | --- | --- | --- | --- |
|  | **Low**  1313 (18.2) | **Medium**  4780 (66.3) | **High**  1118 (15.5) |  |
| **Overweight** | 513 (39.1) | 1902 (39.8) | 487 (43.6) | 0.137 |
| **Obesity-A** | 665 (50.7) | 2427 (50.8) | 532 (47.6) |  |
| **Obesity-B** | 135 (10.3) | 451 (9.4) | 99 (8.9) |  |

SES, socioeconomic status of place of residence

**Table S4.** Mean values for cardiometabolic variables in all subjects

| Risk factor variable and weight category |  | | |
| --- | --- | --- | --- |
|  | **Subjects, n** | **Mean± SD** | **P value** |
| Triglycerides (mg/dL) |  |  | <0.001 |
| Overweight | 2902 | 89**±**42.8 |  |
| Obesity-A | 3624 | 98.6**±**48.5 |  |
| Obesity-B | 685 | 103.1**±**49.7 |  |
| HDL cholesterol (mg/dL) |  |  | <0.001 |
| Overweight | 2902 | 50.9**±**10.8 |  |
| Obesity-A | 3624 | 49.1**±**10.1 |  |
| Obesity-B | 685 | 48.1**±**10.1 |  |
| LDL cholesterol (mg/dL) |  |  | <0.001 |
| Overweight | 2902 | 90.9**±**23.9 |  |
| Obesity-A | 3624 | 93.1**±**24 |  |
| Obesity-B | 685 | 94.7**±**25.3 |  |
| Total cholesterol (mg/dL) |  |  | 0.002 |
| Overweight | 2902 | 159.5**±**29.8 |  |
| Obesity-A | 3624 | 161.4**±**29.1 |  |
| Obesity-B | 685 | 163.4**±**30.2 |  |
| Systolic BP (mmHg) |  |  | <0.001 |
| Overweight | 2059 | 111.8**±**11.8 |  |
| Obesity-A | 2490 | 113**±**12.5 |  |
| Obesity-B | 450 | 114.1**±**12.7 |  |
| Diastolic BP (mmHg) |  |  | 0.003 |
| Overweight | 2059 | 67.6**±**8.7 |  |
| Obesity-A | 2490 | 68.5**±**8.8 |  |
| Obesity-B | 450 | 68.2**±**9.0 |  |
| ALT (U/L) |  |  | 0.001 |
| Overweight | 2902 | 17.6**±**11.2 |  |
| Obesity-A | 3624 | 19.9**±**13.8 |  |
| Obesity-B | 685 | 22.7**±**17.5 |  |

ALT, alanine aminotransferase; BP, blood pressure; HDL, high-density lipoprotein; LDL, low-density lipoprotein

**Table S5.** Odds ratios for cardiometabolic risk factors among adolescents by sex and weight

| Risk factor variable and weight category | All adolescents | | | Female adolescents | | | Male adolescents | | |
| --- | --- | --- | --- | --- | --- | --- | --- | --- | --- |
|  | **Subjects, n** | **Odds ratio, (95% CI)** | **P value** | **Subjects, n** | **Odds ratio, (95% CI)** | **P value** | **Subjects, n** | **Odds ratio, (95% CI)** | **P value** |
| Triglycerides |  |  |  |  |  |  |  |  |  |
| Overweight | 2413 | Reference |  | 1502 | Reference |  | 911 | Reference |  |
| Obesity-A | 2818 | 1.57  (1.32-1.87) | **<0.001** | 1592 | 1.39  (1.11-1.73) | **0.004** | 1226 | 2.01  (1.49-2.70) | **<0.001** |
| Obesity-B | 511 | 1.95  (1.49-2.7) | **<0.001** | 280 | 1.88  (1.31-2.66) | **0.001** | 231 | 2.28  (1.46-3.54) | **<0.001** |
| HDL cholesterol |  |  |  |  |  |  |  |  |  |
| Overweight | 2413 | Reference |  | 1502 | Reference |  | 911 | Reference |  |
| Obesity-A | 2818 | 1.52  (1.20-1.91) | **<0.001** | 1592 | 1.45  (1.03-2.04) | **0.04** | 1226 | 1.56  (1.14-2.15) | **0.005** |
| Obesity-B | 511 | 2.25  (1.59-3.17) | **<0.001** | 280 | 2.59  (1.60-4.20) | **<0.001** | 231 | 1.93  (1.19-3.11) | **0.007** |
| LDL cholesterol |  |  |  |  |  |  |  |  |  |
| Overweight | 2413 | Reference |  | 1502 | Reference |  | 911 | Reference |  |
| Obesity-A | 2818 | 1.21  (0.97-1.51) | 0.09 | 1592 | 1.04  (0.79-1.38) | 0.78 | 1226 | 1.61  (1.10-2.35) | **0.01** |
| Obesity-B | 511 | 1.66  (1.17-2.34) | **0.004** | 280 | 1.63  (1.05-2.52) | **0.03** | 231 | 1.85  (1.05-3.23) | **0.03** |
| Total Cholesterol |  |  |  |  |  |  |  |  |  |
| Overweight | 2413 | Reference |  | 1502 | Reference |  | 911 | Reference |  |
| Obesity-A | 2818 | 1.14  (0.94-1.37) | 0.19 | 1592 | 1.04  (0.83-1.31) | 0.73 | 1226 | 1.4  (0.99-1.96) | 0.051 |
| Obesity-B | 511 | 1.14  (0.82-1.58) | 0.44 | 280 | 1.23  (0.83-1.83) | 0.31 | 231 | 1.06  (0.59-1.91) | 0.85 |
| Systolic BP |  |  |  |  |  |  |  |  | **0.004** |
| Overweight | 1844 | Reference |  | 1153 | Reference |  | 691 | Reference |  |
| Obesity-A | 2151 | 1.74  (1.43-2.18) | **<0.001** | 1190 | 1.69  (1.26-2.28) | **<0.001** | 961 | 1.75  (1.33-2.30) | **<0.001** |
| Obesity-B | 380 | 1.95  (1.41-2.69) | **<0.001** | 208 | 2.16  (1.36-3.43) | **0.001** | 172 | 1.76  (1.13-2.74) | **0.01** |
| Diastolic BP |  |  |  |  |  |  |  |  |  |
| Overweight | 1844 | Reference |  | 1153 | Reference |  | 691 | Reference |  |
| Obesity-A | 2151 | 1.26  (1.06-1.39) | **0.008** | 1190 | 1.19  (0.96-1.48) | 0.12 | 961 | 1.34  (1.02-1.76) | **0.03** |
| Obesity-B | 380 | 1.20  (0.89-1.61) | 0.24 | 208 | 1.29  (0.87-1.89) | 0.20 | 172 | 1.06  (0.66-1.71) | 0.81 |
| Prediabetes |  |  |  |  |  |  |  |  | 0.07 |
| Overweight | 2413 | Reference |  | 1502 | Reference |  | 911 | Reference |  |
| Obesity-A | 2818 | 1.34  (1.11-1.62) | **0.002** | 1592 | 1.33  (1.04-1.7) | **0.02** | 1226 | 1.32  (0.99-1.76) | 0.055 |
| Obesity-B | 511 | 1.71  (1.27-2.29) | **<0.001** | 280 | 1.78  (1.21-2.64) | **0.004** | 231 | 1.56  (0.99-2.44) | 0.052 |
| Diabetes |  |  |  |  |  |  |  |  | 0.11 |
| Overweight | 2413 | Reference |  | 1502 | Reference |  | 911 | Reference |  |
| Obesity-A | 2818 | 0.65  (0.37-1.12) | 0.12 | 1592 | 0.78  (0.39-1.65) | 0.49 | 1226 | 0.47  (0.18-1.22) | 0.12 |
| Obesity-B | 511 | 0.32  (0.08-1.36) | 0.12 | 280 | 0.29  (0.04-2.22) | 0.24 | 231 | 0.36  (0.05-3.77) | 0.32 |
| ALT |  |  |  |  |  |  |  |  |  |
| Overweight | 2312 | Reference |  | 1430 | Reference |  | 882 | Reference |  |
| Obesity-A | 2681 | 1.68  (1.45-1.96) | **<0.001** | 1517 | 1.78  (1.43-2.22) | **<0.001** | 1164 | 1.61  (1.30-1.98) | **<0.001** |
| Obesity-B | 481 | 2.35  (1.86-2.97) | **<0.001** | 263 | 2.26  (1.60-3.20) | **<0.001** | 218 | 2.45  (1.77-3.38) | **<0.001** |

GLMs that controlled for age and sex were used for these analyses. Overweight is the reference group.

A p value < 0.05 was considered significant.

ALT, alanine aminotransferase; BP, blood pressure; FPG, fasting plasma glucose; HbA1c, glycated hemoglobin; HDL, high-density lipoprotein; LDL, low-density lipoprotein

**Table S6.** Odds ratios for cardiometabolic risk factors among children by sex and weight

| Risk factor variable and weight category | All children | | | Girls | | | Boys | | |
| --- | --- | --- | --- | --- | --- | --- | --- | --- | --- |
|  | **Subjects, n** | **Odds ratio, (95% CI)** | **P value** | **Subjects, n** | **Odds ratio, (95% CI)** | **P value** | **Subjects, n** | **Odds ratio, (95% CI)** | **P value** |
| Triglycerides |  |  |  |  |  |  |  |  |  |
| Overweight | 489 | Reference |  | 314 | Reference |  | 175 | Reference |  |
| Obesity-A | 806 | 1.20  (0.79-1.83) | 0.39 | 526 | 1.22  (0.76-1.95) | 0.42 | 280 | 1.21  (0.47-3.08) | 0.69 |
| Obesity-B | 174 | 1.69  (0.95-3.02) | 0.07 | 111 | 1.75  (0.92-3.34) | 0.09 | 63 | 1.60  (0.44-5.77) | 0.47 |
| HDL cholesterol |  |  |  |  |  |  |  |  |  |
| Overweight | 489 | Reference |  | 314 | Reference |  | 175 | Reference |  |
| Obesity-A | 806 | 1.43  (0.65-3.14) | 0.38 | 526 | 1.81  (0.65-5.03) | 0.25 | 280 | 0.94  (0.26-3.38) | 0.92 |
| Obesity-B | 174 | 1.58  (0.52-4.78) | 0.42 | 111 | 1.72  (0.40-7.30) | 0.47 | 63 | 1.4  (0.25-7.84) | 0.70 |
| LDL cholesterol |  |  |  |  |  |  |  |  |  |
| Overweight | 489 | Reference |  | 314 | Reference |  | 175 | Reference |  |
| Obesity-A | 806 | 0.94  (0.60-1.46) | 0.79 | 526 | 0.98  (0.58-1.65) | 0.93 | 280 | 0.87  (0.38-2) | 0.74 |
| Obesity-B | 174 | 1.29  (0.69-2.41) | 0.42 | 111 | 1.27  (0.60-2.67) | 0.52 | 63 | 1.42  (0.47-4.34) | 0.54 |
| Total Cholesterol |  |  |  |  |  |  |  |  |  |
| Overweight | 489 | Reference |  | 314 | Reference |  | 175 | Reference |  |
| Obesity-A | 806 | 0.83  (0.59-1.17) | 0.29 | 526 | 0.79  (0.52-1.19) | 0.27 | 280 | 0.93  (0.48-1.8) | 0.83 |
| Obesity-B | 174 | 1.15  (0.69-1.90) | 0.59 | 111 | 1.05  (0.57-1.92) | 0.87 | 63 | 1.45  (0.59-3.56) | 0.42 |
| Systolic BP |  |  |  |  |  |  |  |  |  |
| Overweight | 215 | Reference |  | 129 | Reference |  | 86 | Reference |  |
| Obesity-A | 339 | 2.10  (1.36-3.24) | **0.001** | 223 | 1.8  (1.05-2.97) | **0.03** | 116 | 2.98  (1.35-6.59) | **0.007** |
| Obesity-B | 70 | 2.29  (1.28-4.11) | **0.005** | 43 | 2.36  (1.18-4.71) | **0.02** | 27 | 2.20  (0.73-6.60) | 0.161 |
| Diastolic BP |  |  |  |  |  |  |  |  | 0.31 |
| Overweight | 215 | Reference |  | 129 | Reference |  | 86 | Reference |  |
| Obesity-A | 339 | 1.44  (0.89-2.34) | 0.14 | 223 | 1.21  (0.67-2.17) | 0.53 | 116 | 2.07  (0.86-4.99) | 0.10 |
| Obesity-B | 70 | 1.75  (0.90-3.41) | 0.09 | 43 | 1.45  (0.63-3.33) | 0.38 | 27 | 2.51  (0.81-7.82) | 0.11 |
| Prediabetes |  |  |  |  |  |  |  |  |  |
| Overweight | 489 | Reference |  | 314 | Reference |  | 175 | Reference |  |
| Obesity-A | 806 | 0.88  (0.47-1.65) | 0.69 | 526 | 0.90  (0.41-1.96) | 0.79 | 280 | 0.83  (0.28-2.43) | 0.73 |
| Obesity-B | 174 | 1.31  (0.55-3.11) | 0.54 | 111 | 1.48  (0.53-4.15) | 0.45 | 63 | 0.92  (0.18-4.69) | 0.92 |
| Diabetes |  |  |  |  |  |  |  |  |  |
| Overweight | 489 | Reference |  | 314 | Reference |  | 175 | Reference |  |
| Obesity-A | 806 | 0.61  (0.15-2.43) | 0.49 | 526 | 0.20  (0.02-1.93) | 0.16 | 280 | 1.88  (0.19-18.26) | 0.58 |
| Obesity-B | 174 | 0 | 1 | 111 | 0 | 1 | 63 | 0 | 1 |
| ALT |  |  |  |  |  |  |  |  |  |
| Overweight | 471 | Reference |  | 305 | Reference |  | 166 | Reference |  |
| Obesity-A | 759 | 1.61  (1.19-2.18) | **0.002** | 490 | 1.72  (1.20-2.46) | **0.003** | 269 | 1.39  (0.78-2.47) | 0.26 |
| Obesity-B | 165 | 2.45  (1.61-3.72) | **<0.001** | 105 | 2.25  (1.36-3.73) | **0.002** | 60 | 2.98  (1.42-6.25) | **0.004** |

GLMs that controlled for age and sex were used for these analyses. Overweight is the reference group.

A p value < 0.05 was considered significant.

ALT, alanine aminotransferase; BP, blood pressure; FPG, fasting plasma glucose; HbA1c, glycated hemoglobin; HDL, high-density lipoprotein; LDL, low-density lipoprotein

**Table S7:** Clustering of cardiometabolic risk factors among children and adolescents by weight category

| Weight category | Number of cardiometabolic risk factors  prevalence, % | | | | P value |
| --- | --- | --- | --- | --- | --- |
|  | **0** | **1** | **2** | **≥3** | **<0.001** |
| Overweight | 57.4 | 31.7 | 8.4 | 2.5 |  |
| Obesity-A | 47.7 | 34.1 | 14.2 | 4 |  |
| Obesity-B | 42 | 33.6 | 17.8 | 6.6 |  |

Cardiometabolic risk factors include hyperglycemia (prediabetes or diabetes), high triglycerides, LDL cholesterol, ALT, and systolic or diastolic BP or low HDL cholesterol. ALT, alanine aminotransferase; BP, blood pressure; HDL, high-density lipoprotein; LDL, low-density lipoprotein

1. **Supplementary Figures**

Children and adolescents aged 5-17 years with a documented BMI of ≥ 85th percentile during

Jan 2020 - May 2021

n=26783

Subjects excluded because of missing/incomplete lipid profile during Jan 2020 - May 2021, or

BMI≥120% of the 95th percentile

n=19572

Subjects included in this analysis

n=7211

**Figure S1**. CONSORT diagram of subjects included in the cross-sectional analysis

**
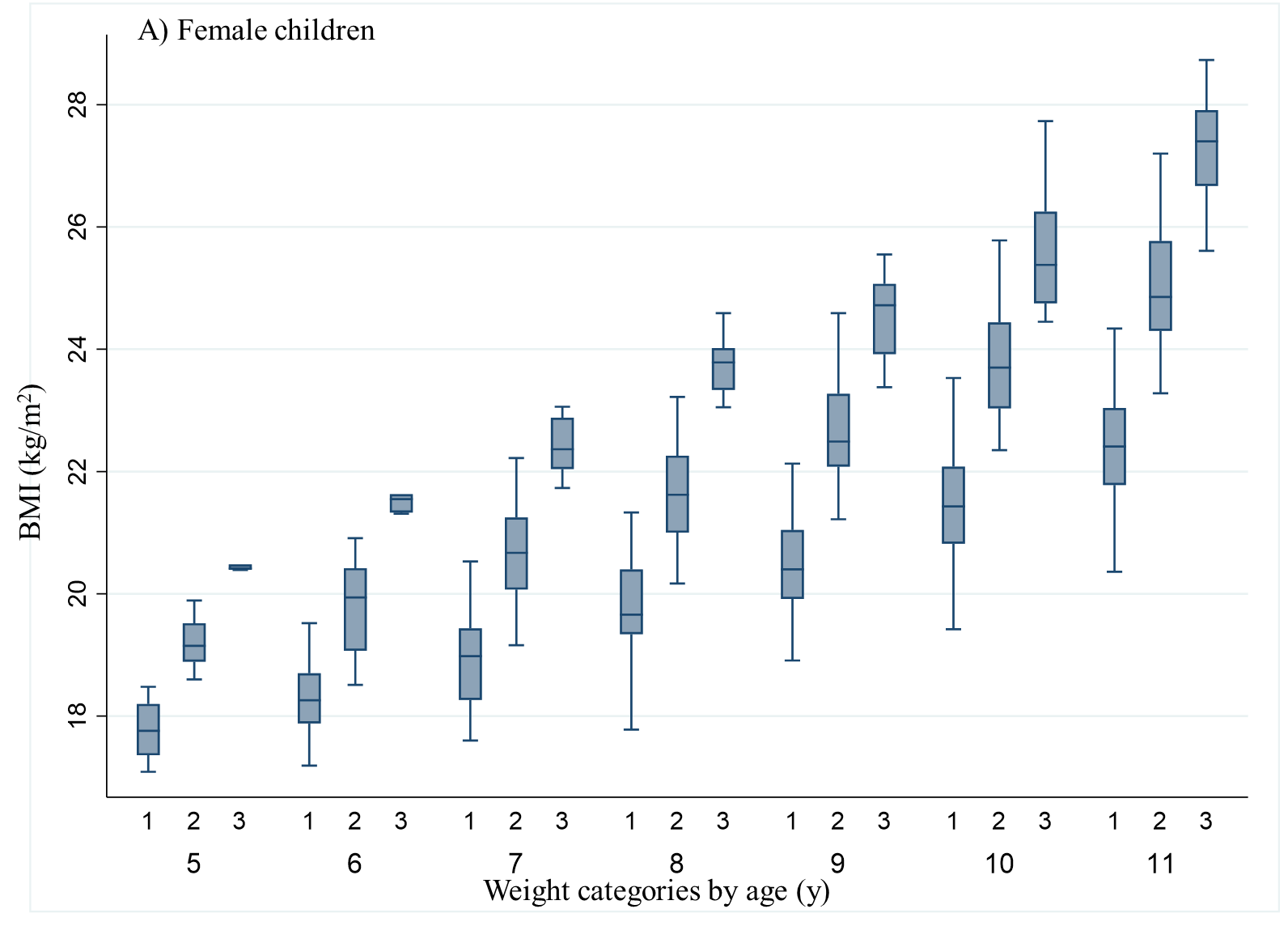
**

**
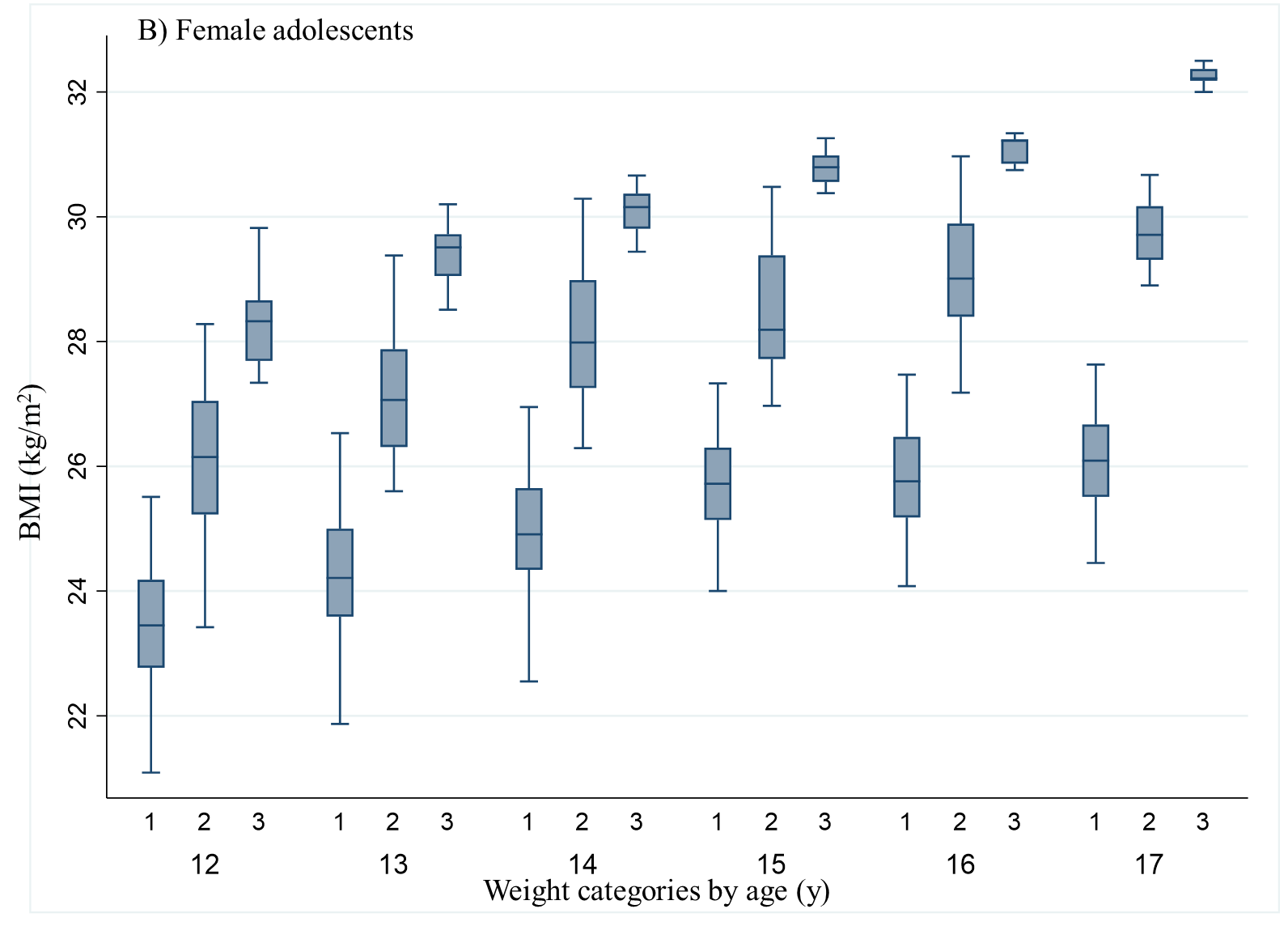
**

**
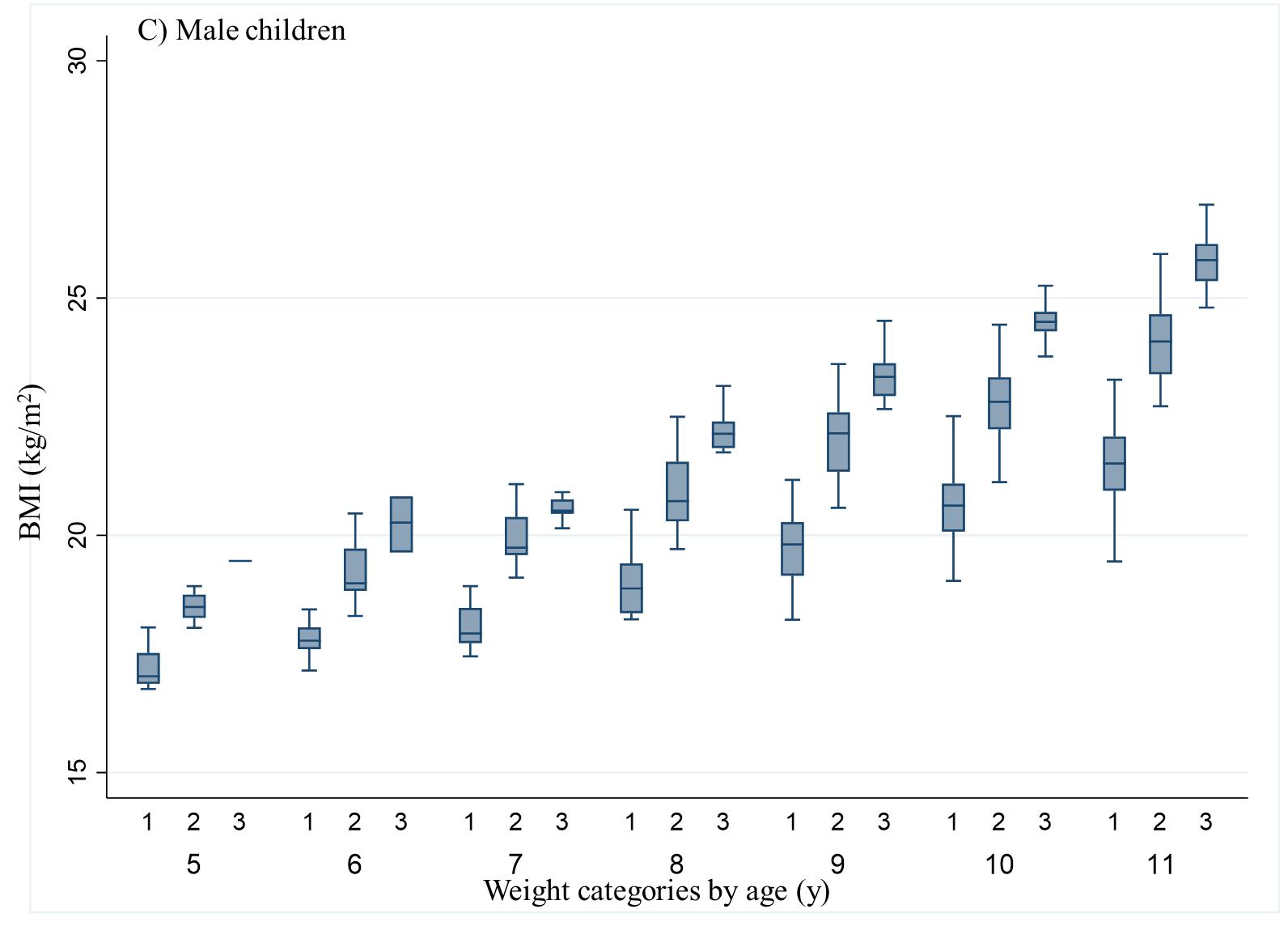
**

**
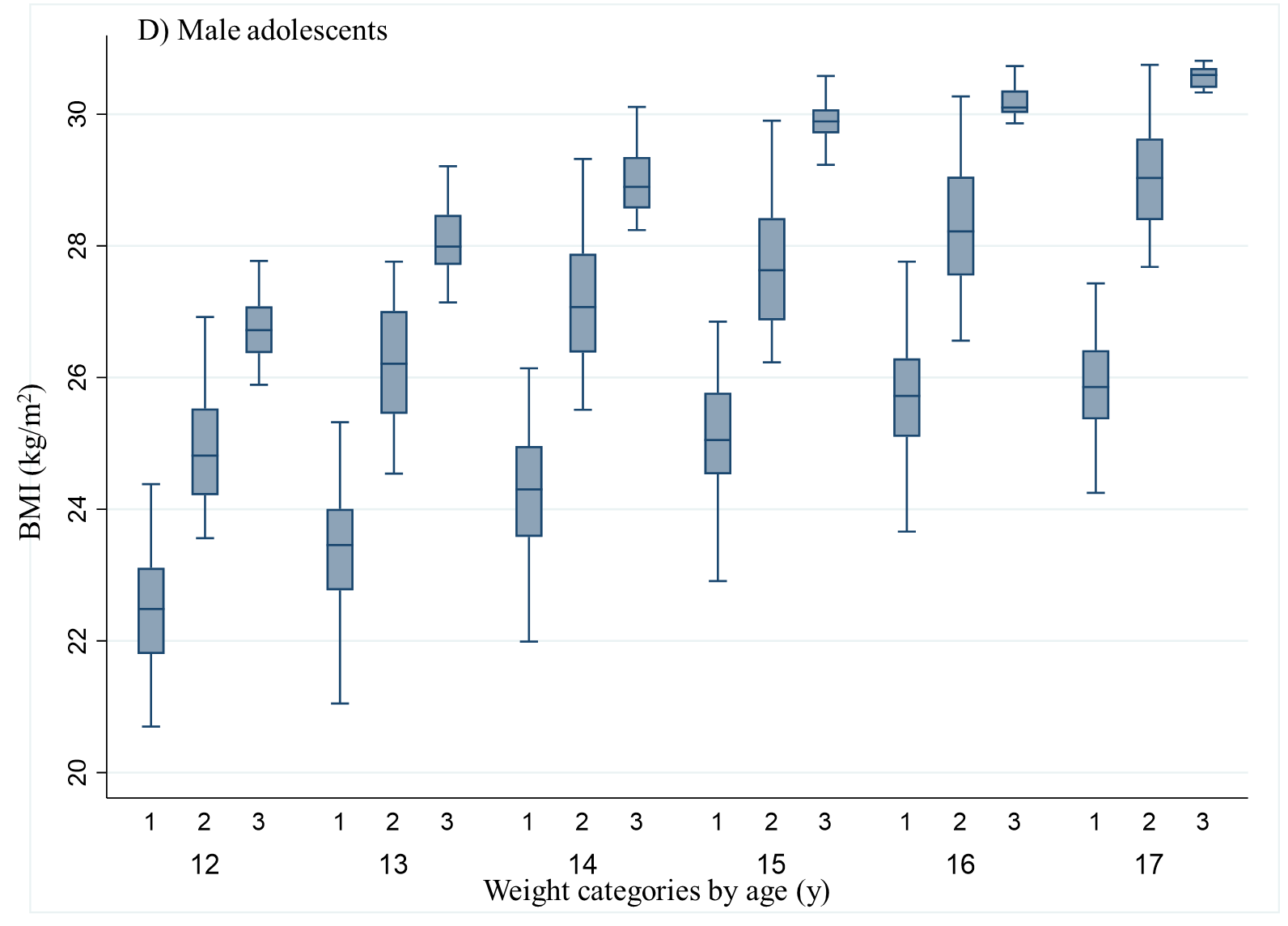
**

**Figure S2.** Median BMI values classified by weight categories and age: a) female children, b) female adolescents, c) male children, and d) male adolescents**.** Figures show a box plot of BMI values (kg/m^2^) by weight categories (1=overweight; 2=obesity-A; 3=obesity-B) and age (y). In the box plot, the black horizontal bars indicate medians; the tops and bottoms of the boxes the upper and lower quartiles, respectively; and the whiskers indicate the smallest or highest values.
